# Nanocrystallization in FINEMET-Type Fe_73.5_Nb_3_Cu_1_Si_13.5_B_9_ and Fe_72.5_Nb_1.5_Mo_2_Cu_1.1_Si_14.2_B_8.7_ Thin Films

**DOI:** 10.3390/ma13020348

**Published:** 2020-01-12

**Authors:** Evgeniya A. Mikhalitsyna, Vasiliy A. Kataev, Aitor Larrañaga, Vladimir N. Lepalovskij, Galina V. Kurlyandskaya

**Affiliations:** 1 Department of Solid State Magnetism IPAM, Ural Federal University, Mira st. 19, 620002 Ekaterinburg, Russia; evgenia.mihalitsyna@urfu.ru (E.A.M.); vakataev@urfu.ru (V.A.K.); vladimir.lepalovsky@urfu.ru (V.N.L.); 2 SGIKER, Servicios Generales de Investigación, Universidad del País Vasco UPV-EHU, Apartado 644, 48080 Bilbao, Spain; aitor.larranaga@ehu.eus; 3 Departamento de Electricidad y Electrónica, Universidad del País Vasco UPV-EHU and BCMaterials, Apartado 644, 48080 Bilbao, Spain

**Keywords:** thin magnetic film, annealing treatment, FINEMET, soft magnetic material, X-ray diffraction, sensor applications

## Abstract

A growing variety of microelectronic devices and magnetic field sensors as well as a trend of miniaturization demands the development of low-dimensional magnetic materials and nanostructures. Among them, soft magnetic thin films of Finemet alloys are appropriate materials for sensor and actuator devices. Therefore, one of the important directions of the research is the optimization of thin film magnetic properties. In this study, the structural transformations of the Fe_73.5_Nb_3_Cu_1_Si_13.5_B_9_ and Fe_72.5_Nb_1.5_Mo_2_Cu_1.1_Si_14.2_B_8.7_ films of 100, 150 and 200 nm thicknesses were comparatively analyzed together with their magnetic properties and magnetic anisotropy. The thin films were prepared using the ion-plasma sputtering technique. The crystallization process was studied by certified X-ray diffraction (XRD) methods. The kinetics of crystallization was observed due to the temperature X-ray diffraction (TDX) analysis. Magnetic properties of the films were studied by the magneto-optical Kerr microscopy. Based on the TDX data the delay of the onset crystallization of the films with its thickness decreasing was shown. Furthermore, the onset crystallization of the 150 and 200 nm films began at the temperature of about 400–420 °C showing rapid grain growth up to the size of 16–20 nm. The best magnetic properties of the films were formed after crystallization after the heat treatment at 350–400 °C when the stress relaxation took place.

## 1. Introduction

Amorphous and nanocrystalline soft magnetic (SM) alloys are important materials attracting both applied and fundamental interest. The main property of soft ferromagnets is a low value of coercivity (H_C_). The low H_C_ level, as well as high values of a saturation induction and a relative magnetic permeability, identify the SM alloys application prospects in electric motors, transformers and highly sensitive magnetic field detectors [1,2,3]. The functional properties of SM alloys can be controlled by means of heat treatments over a wide temperature range, thermomechanical and thermomagnetic treatments based on the requirements for particular devices [4]. For example, we can mention nanocrystalline materials magnetoimpedance effect based sensors, which have very high sensitivity with respect to external applied magnetic field and very good time stability in certain conditions in comparison with amorphous alloys [5].

Among the soft magnetic materials alloys based on the Fe–M–Cu–Si–B system, where M is a transition metal, which is one of the most well-known [6,7,8,9]. The Finemet alloy (Fe_73.5_Nb_3_Cu_1_Si_13.5_B_9_) has gained the most popularity because of its soft magnetic properties: high saturation flux density, excellent temperature characteristics, low core loss and magnetostriction and high permeability [10,11]. Advantages of the Finemet-type alloys allow being used for energy-saving, volume reduction, high magnetic performance, noise reduction and high frequency and sensory application [3,12]. These alloys are usually produced as ribbons by the melt-spinning technique. The excellent soft magnetic properties of the Finemet-type ribbons are governed by the special nanostructural state that is 10–15 nm grains dispersed in a residual amorphous matrix [12,13,14,15], which is achieved in the course of heat treatment of the initially amorphous ribbons. The above-mentioned properties are directly related to the averaging of magnetocrystalline anisotropy via the exchange interactions between grains [16]. The implementation of similar Finemet alloy properties in the thin film state would allow developing a wide range of miniature devices requested for special conditions like corrosive environments. For instance, the Finemet-type thin films can be considered as appropriate materials for the elements of sensor devices [13,17,18]. However, the sputtering conditions, film composition, effects of film thickness and a substrate are additional parameters determining nanostructure of the alloys and their magnetic properties subsequently. As well defined uniaxial magnetic anisotropy with low local anisotropy axes distribution is very important for many sensitive elements response, Ni, Cr, Mo doped Fe–M–Cu–Si–B systems seems to be interesting for particular applications [4,5]. Above-mentioned dopants are playing a special role in the induced magnetic anisotropy formation processes [5].

For the SM films, heat treatment is also an eligible option for the grain sizes regulation and the internal stresses diminution [19,20,21]. Herewith the heat treatment conditions can differ from ones for ribbons and should be examined along with nanostructural transformations in films during the annealing [22,23,24]. Understanding of the role of structural features and mechanisms of formation of the magnetic uniaxial magnetic anisotropy in the films with a narrow distribution of local easy magnetization axes (EMA) can be the key points to succeed in the achieving of the desired magnetic properties. In particular, well defined transverse magnetic anisotropy is a crucial request for the high magnetoimpedance effect for magnetic field sensors development [25]. It is known, that niobium can be partially replaced by Cr or Mo aiming to improve the soft magnetic properties [26] and further reduce costs. The Finemet-type ribbons and wires structure with various inhibitor atoms has been carefully analyzed. In spite of this, structural transformations of the thin films, as well as the effect of thickness on the structure, are still not well understood.

In this work, the amorphous-to-nanocrystalline transformation of the Fe_73.5_Nb_3_Cu_1_Si_13.5_B_9_ and Fe_72.5_Nb_1.5_Mo_2_Cu_1.1_Si_14.2_B_8.7_ films were studied and analyzed with their magnetic properties. Thus, the relationship between nanostructure and magnetic properties is defined with the view of the determination of the optimal soft magnetic properties of the films.

## 2. Materials and Methods 

The thin films with thicknesses of 100, 150 and 200 nm and with nominal compositions of Fe_73.5_Nb_3_Cu_1_Si_13.5_B_9_ and Fe_72.5_Nb_1.5_Mo_2_Cu_1.1_Si_14.2_B_8.7_ were prepared using a high-frequency ion-plasma sputtering technique in argon onto. The background pressure in the chamber was as high as 5 × 10^−7^ mbar. The working Ar pressure was 10^−4^ mbar. The constant magnetic field of about 8 kA/m was applied during the deposition in the film plane with the aim of induced uniaxial magnetic anisotropy formation. The magnetic field was created by specially designed system of the permanent magnets. The targets of above mentioned compositions were used for the films deposition and the obtained compositions were additionally checked after the deposition. The deposition rate was about 28 nm/min. The borosilicate glass (Wilmad LabGlass©, Vineland, NJ, USA) and (110) monocrystalline silicon covered with SiO_2_ layer were used as the substrates. The substrate temperature during the deposition was checked using additional calibration, it was close to 40–50 °C.

The thin films structural state was modified by the heat treatments. The heating was performed in vacuum in the sputtering chamber. Heating was done using an infrared lamp. The temperature was measured near the film surface using a thermocouple. The range of established annealing temperature fluctuations did not exceed ±5 °C.

The thin films thicknesses were verified by the height difference between the film and the substrate by Dektak 150 Stylus Profilometer (Veeco, Somerset, NJ, USA) using a specially formed on the substrate sharp step.

The thin film’s structure was investigated by means of a set of XRD methods. (i) The Fe_73.5_Nb_3_Cu_1_Si_13.5_B_9_ thin film samples with the thickness of 200 nm were investigated by Bruker D8 Discover diffractometer with the Cu-Kα radiation (the radiation wavelength was λ = 1.5418 Å) and a graphite monochromator. The measurements were carried out in the 2Θ angle range from 10 to 125° at the room temperature. The XRD patterns were processed using the TOPAS 3 program with the Rietveld algorithm for the refinement of structural parameters, and the crystallite size was determined from the XRD patterns by the estimation of peak broadening at half its height using the Selyakov–Scherrer formula [27,28]. (ii) A common XRD analysis of the Fe_72.5_Nb_1.5_Mo_2_Cu_1.1_Si_14.2_B_8.7_ films was carried out using PHILIPS X’PERT PRO diffractometer with the Cu–Kα radiation (the radiation wavelength was λ = 1.5418 Å). Data were collected at the room temperature in the 2Θ angle range from 35 to 70° with a step of 0.026°. An anti-scattering slit with a fixed deflection was used for a constant sample illumination area. (iii) A high-temperature XRD was performed in-situ for the films with thicknesses of 100, 150 and 200 nm by means of Bruker D8 Advance Vantec diffractometer with the Cu–Kα radiation (the radiation wavelength was λ = 1.5418 Å) equipped with Vantec-1 PSD detector and Anton Paar HTK2000 high-temperature heater. The measurements were carried out in the 2Θ angle range from 35 to 66° at the temperatures of up to 700 °C with a step of 5 °C for the films with the thicknesses of 100 and 200 nm and with a step of 10 °C for the film with the thickness of 150 nm. The heating rate was 0.33 °C/s, and the measurement time of the XRD pattern at each temperature was approximately 18 min.

A surface study was carried out in the tapping mode on a scanning probe microscope (SPM) Multimode 8 (Bruker, Santa Barbara, CA, USA). The scan area was 10 × 10 μm^2^ (resolution 512 pixels × 256 pixels) and the scan rate was 3 μm/s, the tip radius was about 40 nm and spring constant −5 N/m.

Temperature dependences of magnetization were measured using the magnetic properties measurement system MPMS XL7. An applied magnetic field during the measurement process was 8 kA/m. The magnetic hysteresis loops of the films underwent various heat treatments were investigated by means of a magneto-optical microscope Evico Magnetics GmbH for the external magnetic field (H) applied in plane of the film and in the direction of the easy magnetization axis (EMA) defined during the experimental measurements of the angular dependences of magnetization M, i.e., M(H) curves.

## 3. Results and Discussion

**Figure 1:**
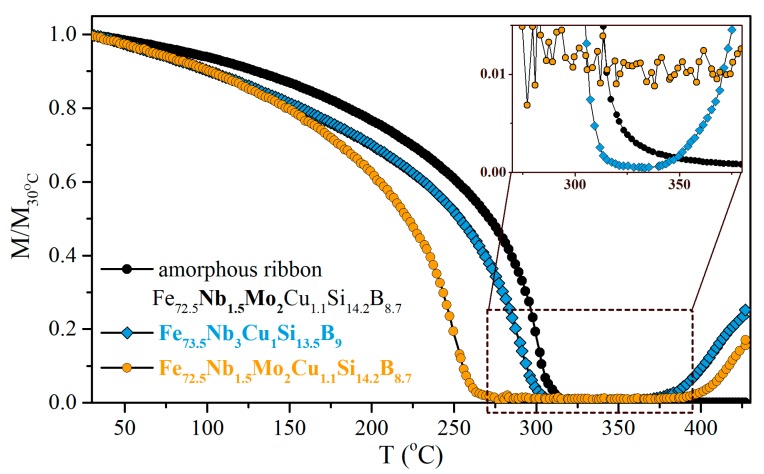
M vs. T dependences of Fe_72.5_Nb_1.5_Mo_2_Cu_1.1_Si_14.2_B_8.7_ and Fe_73.5_Nb_3_Cu_1_Si_13.5_B_9_ films with the thickness of 200 nm and amorphous ribbon Fe_72.5_Nb_1.5_Mo_2_Cu_1.1_Si_14.2_B_8.7_ for the Curie temperature evaluation. The inset shows the transition region with better resolution.

The magnetic properties of the studied thin films critically depend on their structure. Initial information about the structural transformations was obtained from the analysis of the temperature dependences of magnetization. Figure 1 shows the relative magnetization dependences (M/M_30°C_) on the temperature of the Fe_72.5_Nb_1.5_Mo_2_Cu_1.1_Si_14.2_B_8.7_ and Fe_73.5_Nb_3_Cu_1_Si_13.5_B_9_ thin films with the thickness of 200 nm. The obtained curves allow analyzing the features of a new structure emergence during the amorphous-to-nanocrystalline transition. For comparison, the temperature dependence of magnetization of the Fe_72.5_Nb_1.5_Mo_2_Cu_1.1_Si_14.2_B_8.7_ amorphous ribbon is also shown. The nature of the dependences is identical qualitatively, but there is a difference in critical temperatures. Figure 1 shows the temperature dependences of magnetizations measured in order to define the Curie temperatures (T_C_) of thin films. As to expect due to finite size effects, T_C_ of the as-deposited films is lower in comparison with the amorphous ribbon along with a markedly lower temperature at which crystallization begins. T_C_ of the amorphous phase changes with variations of the inhibitor/doping elements in the film composition as T_C_(Nb) > T_C_(NbMo). The higher Curie temperature was observed for Fe_73.5_Nb_3_Cu_1_Si_13.5_B_9_ in comparison with Fe_72.5_Nb_1.5_Mo_2_Cu_1.1_Si_14.2_B_8.7_ one. Furthermore, in the temperature range at which the amorphous phase of the films is paramagnetic, a non-zero value of magnetization is observed: the largest for the Fe_72.5_Nb_1.5_Mo_2_Cu_1.1_Si_14.2_B_8.7_ film (inset for Figure 1). The magnetization begins to increase above 380 °C, indicating the formation of significant amount of a new (crystalline) phase of the films.

### 3.1. Heat Treatment Influence on the Fe_73.5_Nb_3_Cu_1_Si_13.5_B_9_ Thin Film Structure

**Figure 2:**
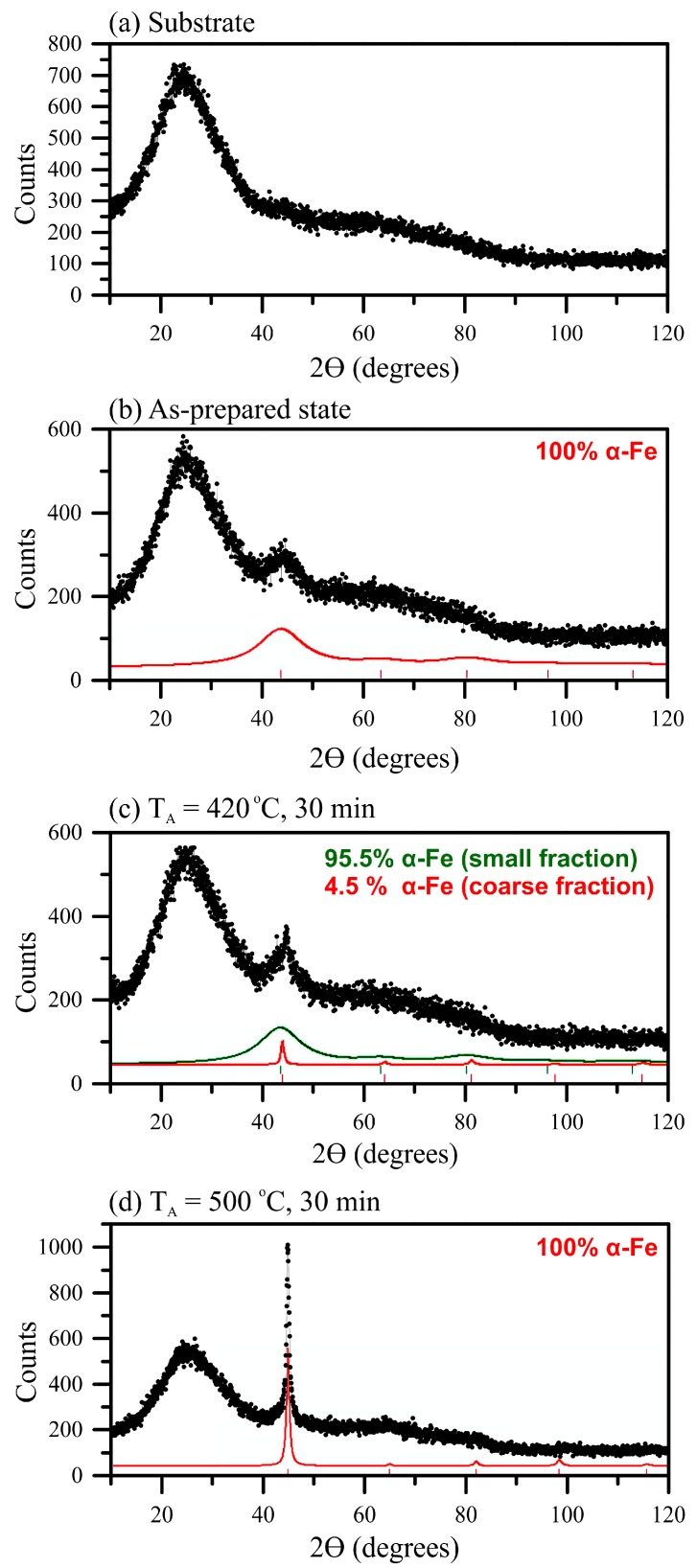
XRD data of the glass substrate (**a**) and Fe_73.5_Nb_3_Cu_1_Si_13.5_B_9_ film with the thickness of 200 nm in the as-prepared state (**b**) and after the heat treatment at the temperatures of 420 °C (**c**) and 500 °C (**d**) for 30 min.

Based on the data obtained from the temperature dependences of magnetization, a series of heat treatments of the 200 nm thin films were carried out. The films were annealed at the temperatures of 420 and 500 °C for 30 min in order to obtain different structural states. For the films of classic composition (Fe_73.5_Nb_3_Cu_1_Si_13.5_B_9_) a standard X-ray diffraction analysis was performed. The XRD patterns of the as-prepared and annealed films are shown in Figure 2.

In view of the small film thickness the intensity of peak, which appears in the case of the XRD pattern of the as-prepared film in the range of angles of 40–50° (Figure 2b), is almost at the signal level of the substrate (Figure 2a). The signal from the substrate was subsequently subtracted from all XRD patterns. Conclusions about the development of the crystallization process were drawn based on the shape of the peak and peak position changes observed for the XRD patterns of films in the initial state and after the heat treatments. As the heat treatment temperature increased, the appearance of a narrower peak with low intensity was observed from a very wide peak in the same angle range (Figure 2c) that indicates the emergence of a nanocrystalline phase. The intensity of the peak rose with a further increase in temperature (Figure 2d). The average grain size of the crystalline phase was determined as a result of the XRD patterns processing and was analyzed using the Selyakov–Scherrer method for the main peak (110) corresponding to the bcc-Fe(Si) phase. In Figure 2 the percentage of different sizes grains is given only in relation to the entire crystalline phase without taking into account the amorphous component. Since the signal from the amorphous phase is at the signal level of the substrate, it is not possible to estimate the volume of “amorphous phase–crystalline phase” transformation.

The structural state of the as-prepared Fe_73.5_Nb_3_Cu_1_Si_13.5_B_9_ film can be determined as amorphous from the point of view XRD. A diffuse peak in the angle range of 2Θ from 40 to 50° is mainly formed by coherent scattering regions, the size of which is about 1 nm (Figure 2b). Despite the fact that these regions are difficult to be considered structurally ordered, in the further discussion they will be considered as ultrafine grains, which predominantly constitute an “amorphous” matrix of the film. The heat treatment at the temperature of 420 °C leads to the formation of grains with the size of 16 ± 5 nm that are α-Fe(Si) nanocrystallites. Herewith the grains volume fraction in the crystalline phase of the film is only 5%. The main part of the crystalline phase (95%) is still composed of the ultrafine grains about 1 nm in size. After the heat treatment at 500 °C, the crystalline phase consists almost entirely of the grains with the size of about 20 nm. However, the slightly blurred base of the main peak in Figure 2d allows consideration about the presence of the residual fine-grained “amorphous” matrix.

A sharp change in the structural state of the films between the heat treatments at 420 and 500 °C was observed. The films were annealed at a temperature of 420 °C for 30 min (Table 1). To study the temperature range between 420 and 500 °C in details the films of selected thicknesses were annealed at temperature of 450 °C for 10, 20 and 30 min. In the thin films annealed at 450 °C, the crystalline phase is bimodal, i.e., it contains both ultrafine and large grains. The size of the ultrafine grains is about 2 ± 1 nm and it shows no change in course of annealing time. However, the volume fraction of grains in the crystalline phase increases from about 11 to about 20 (Table 1).

Table 1 shows the volume fraction of the large grains increase from 5 to about 20% with an increase in the annealing temperature from 420 to 450 °C when the annealing time is 30 min. When the annealing time changes from 10 to 30 min at the temperature of 450 °C the large grains size slightly increases from 16 to 20 nm but this increase is within the margin of error. The large grains fraction in the crystalline phase rises from about 11 to about 20% corresponding to the changes in the average grain size. Thus, at the given temperature, the volume fraction of the large grains may increase due to the growth of their size. However, the absence of the crystalline phase fraction rise with the increase in the holding time from 20 to 30 min indicates that at the temperature of 450 °C only a small fraction of the possible crystallization centers becomes activated.

The increase of the heat treatment temperature up to 500 °C leads to the structure the crystalline phase of which consists of the large grains of about 20 nm in size, and ultrafine grains are no longer detectable. Despite the fact that this temperature is almost 100 °C higher than the temperature of the onset of crystallization, there are no grains in the structure that are larger than grains arising at the beginning of crystallization. Thus, heating to 500 °C leads to thermal activation of nearly all crystallization centers, and the Nb atoms in the composition effectively play the role of an inhibitor of grain growth as well as in the ribbons. It should be noted that the grains grow up to the characteristic size of approximately 20 nm. Rapid capture of the film volume does not allow grains to grow further, as was observed with an increase in the holding time of annealing at the temperature of 450 °C. Possibly, a small film thickness plays its role since in the direction perpendicular to the film surface a small amount of grains is placed and the contact between the crystalline boundaries can be achieved quite quickly.

### 3.2. Kinetics of Crystallization in Fe_72.5_Nb_1.5_Mo_2_Cu_1.1_Si_14.2_B_8.7_ Thin Films

The temperature X-ray diffraction (TDX) analysis was performed in situ in the diffraction angles range including XRD reflection intervals of the expected crystalline phases. The temperature range from 450 to 650 °C was chosen in order to study a film crystalline structure transformation from the initial to later stages of crystallization. The thin films of different thicknesses (100, 150 and 200 nm) were deposited onto the Si substrate having higher temperature resistance comparing to glass.

**Figure 3:**
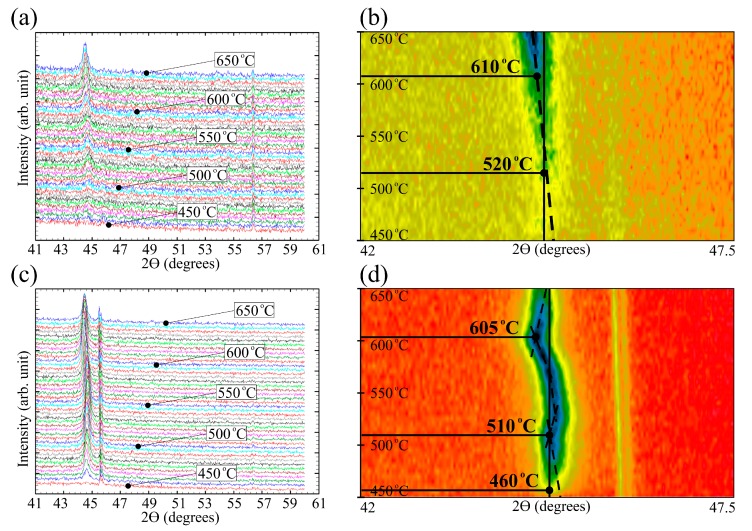
Temperature X-ray diffraction (TDX) data of Fe_72.5_Nb_1.5_Mo_2_Cu_1.1_Si_14.2_B_8.7_ films with thicknesses of 100 (**a**,**b**) and 200 nm (**c**,**d**). XRD patterns at the temperature interval 450–650 °C, step–5 °C (**a**,**c**). View from above on the XRD patterns (**b**,**d**).

Figure 3 shows XRD patterns obtained in the temperature range from 450 to 650 °C for the 100 and 200 nm films (temperature step is 5 °C; average measurement time at the given temperature is 18 min). For both films, an increase in the peaks’ intensity in the region of 43–46° was observed with the annealing temperature increase. It indicates the development of the primary crystallization process and the increase in the crystalline phase volume. It is most clearly seen on the projections of XRD patterns onto a plane (top view), for which the peaks intensity is related to color (Figure 3b,d). Figure 3 shows that thickness of the film significantly affects the course of the crystallization process. For the 100 nm film (Figure 3a,b) the peak indicating the appearance of a crystalline phase are initially poorly resolved at the noise level as well the peak’s height increases evenly with the increase of the temperature up to about 630 °C. In the case of 200 nm film, the first peak was observed at 450–460 °C. The peak height substantially exceeded the peak intensity of the thinner film, and its intensity increased rather quickly with the growth of temperature and reached values close to the maximum at the temperature of about 500 °C.

A peak observed in the angle region of 56° (more clearly for the 100 nm film) over the entire temperature range corresponded to a signal from a silicon substrate. In addition, a peak of lower intensity was observed near the main peak that is seen in the TDX patterns in Figure 3b,d. This peak corresponds to the signal from the platinum substrate on which the sample was placed for the high-temperature studies. For the film with the thickness of 100 nm, the signal from the platinum substrate was hardly noticeable due to the large incoherent scattering of X-rays. The TDX patterns analysis allows drawing conclusions about the kinetics of the average grain size and the nanocrystals lattice parameter changes with the increase in the temperature. However, the processing of the 100 nm film TDX data in the temperature range of 450–480 °C did not provide sufficiently reliable information about the average grain size due to the weak development of the crystallization process. The average grain size can be estimated starting from the temperature 480 °C (the grain size of about 20 nm). With the further temperature increase up to 650 °C, the average grain size keeps being close to 20 nm.

**Figure 4:**
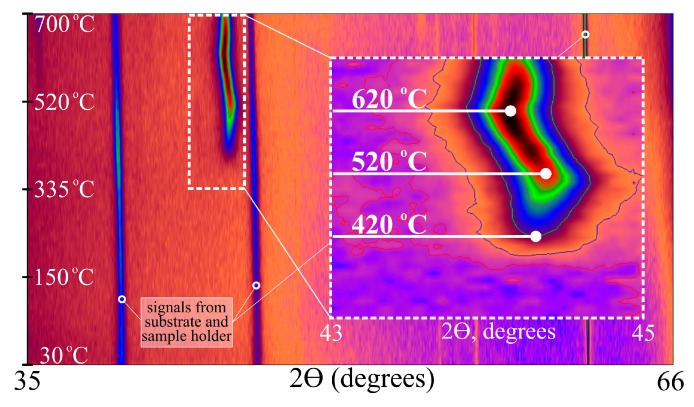
TDX data of Fe_72.5_Nb_1.5_Mo_2_Cu_1.1_Si_14.2_B_8.7_ films with thicknesses of 150 nm.

The determination of the 200 nm film average grain size performed on the main peak (well distinguishable at the temperature of 455 °C) gives the size of about 20 nm and with the temperature increase and hereinafter remains almost unchanged. A significant difference in the crystallization kinetics disclosed for the films with the thicknesses of 100 and 200 nm conditions the need for its more detailed analysis. In this connection, a similar study was performed for the film with the thickness of 150 nm. The angular dependences of the X-rays intensity were obtained in the temperature range from 30 to 700 °C with a step of 10 °C. Figure 4 shows set of projections of the XRD patterns for this film.

The absence of contrast in the 2θ angle range from 43 to 46° and in the temperature range of 30–400 °C (Figure 4) corresponds to the X-ray amorphous state of the film taking into account the presence of the coherent scattering regions of no more than 2 nm in size. Crystallization of the film begins already at the temperature of 400 °C and it is characterized by a sharp grain growth in the temperature range from 400 to 420 °C. The size of the formed grains is about 20 nm. With a further increase in temperature, the grain size increases very little up to slightly above 20 nm. A more detailed analysis of the main peak intensity change shows that the sharpest increase in the peak intensity occurred at the temperature of 520 °C, indicating the increase in crystalline phase volume (in the absence of texture). After that, the peak intensity almost did not change up to 670–680 °C that may indicate a full crystallization of the sample.

Based on the results of the texture analysis for the 200 nm film annealed at 550 °C, and taking into account similar results obtained for the Finemet-type ribbons [29,30] the absence of the texture was assumed. The texture analysis showed a random orientation of the grains during the crystallization process in the Fe_72.5_Nb_1.5_Mo_2_Cu_1.1_Si_14.2_B_8.7_ film with the thickness of 200 nm. As a result, there was no contrast on the pole figures, which indicated the absence of texture in the films.

**Figure 5:**
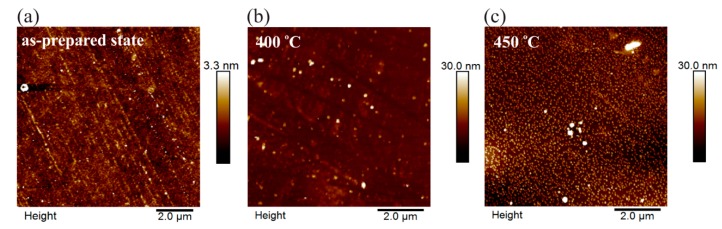
Atomic force microscopy (AFM) patterns of the Fe_73.5_Nb_3_Cu_1_Si_13.5_B_9_ films with thickness of 200 nm in the as-prepared state (**a**) and after the annealing (**b**,**c**).

It should be noted that the presence of the additional element (Mo) in the film composition leads to slightly larger average characteristic size of crystalline formations (2 nm) in the initial state compared with the film composition containing only Nb. The choice of the film composition, its intermediate thickness and careful measurements regime (at lower temperatures) made it possible to determine the crystallization start temperature of Finemet-type films more accurately. In addition to the XRD techniques surface study and magnetic mapping were carried out with scanning probe microscopy. More details on this technique can be found elsewhere [31,32]. Surface topography of the 200 nm films in the as-prepared state and after annealing at 400 and 450 °C are presented in Figure 5. The surface of the films in the initial state and after the heat treatment up to 400 °C is quite smooth and without any pronounced features. According to the XRD data the heat treatment at the temperature of 450 °C leads to the crystallization of the film. AFM (atomic force microscopy) data shows the formation of the grain-like structure on the film’s surface. The grains size was about 60–90 nm in diameter and 7–10 nm in height. The root mean square roughness was about 0.5, 1.6 and 4.0 nm for as-prepared film and films annealed at 400 and 450 °C accordingly.

**Figure 6:**
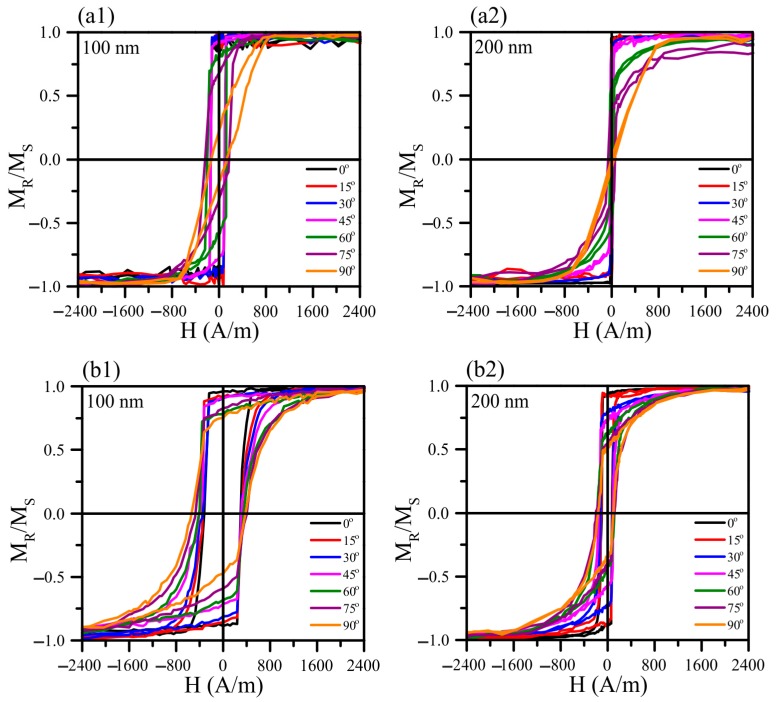
Hysteresis loops of the of Fe_73.5_Nb_3_Cu_1_Si_13.5_B_9_ (**a1**,**a2**) and Fe_72.5_Nb_1.5_Mo_2_Cu_1.1_Si_14.2_B_8.7_ (**b1**,**b2**) films with thicknesses of 100 and 200 nm in the as-prepared state measured under different angles with respect to the direction of the magnetic field applied during deposition: zero angle coincides with the direction of the easy magnetization axis (EMA).

The magnetic properties of the heat-treated Fe_73.5_Nb_3_Cu_1_Si_13.5_B_9_ and Fe_72.5_Nb_1.5_Mo_2_Cu_1.1_Si_14.2_B_8.7_ films with the thickness of 10–200 nm were studied using the magneto-optical Kerr microscopy in the direction of the external magnetic field applied along the easy magnetization axis, i.e., in the same direction as that one of the external magnetic field applied during thin film deposition. The angle (Figure 6) was determined as the angle between the magnetic field during the measurement and the direction of the magnetic field applied during the sample preparation. 

The presence of the magnetic field during the deposition of the films has led to the induced magnetic anisotropy formation with the easy magnetization axis coincident to the field direction. The hysteresis loop is rectangular, and the coercive force is minimal along the EMA. In the direction perpendicular to the easy magnetization axis, the hysteresis loop has the inclined shape.

**Figure 7:**
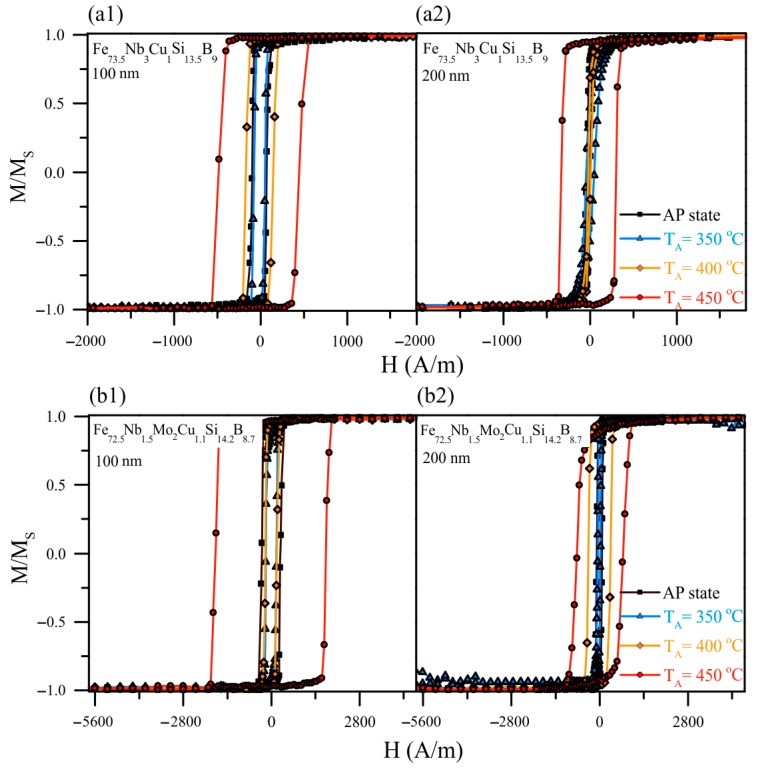
Hysteresis loops of the of Fe_73.5_Nb_3_Cu_1_Si_13.5_B_9_ (**a1**,**a2**) and Fe_72.5_Nb_1.5_Mo_2_Cu_1.1_Si_14.2_B_8.7_ (**b1**,**b2**) films with thicknesses of 100 and 200 nm in the as-prepared state and after annealing measured in the direction of the EMA.

Figure 7 shows hysteresis loops of the 100 and 200 nm films for comparative analysis of the data obtained from the XRD and TDX studies. It is seen that the hysteresis loop shape and the coercivity of the films undergo changes as a result of the heat treatment, as well as depending on the thickness and chemical composition of the samples. Regardless of the thickness and composition, the coercivity of the films decreased and the shape of the hysteresis loops became less rectangular after the heat treatment at 350 °C. With a subsequent increase in temperature, both an increase and a decrease in the coercivity were observed depending on the type of the film. After the heat treatment at 450 °C, the coercivity of all of the films increased dramatically. The coercivity values as a function of the annealing temperature and composition are presented in Table 2. It is clearly seen the elevated value of coercivity of the Fe_72.5_Nb_1.5_Mo_2_Cu_1.1_Si_14.2_B_8.7_ films in comparison with Fe_73.5_Nb_3_Cu_1_Si_13.5_B_9_ films.

A qualitative similarity of the temperature dependencies of magnetization of the films and the ribbon (Figure 1) indicates an identity of the processes occurring in them under the same heating conditions. However, the Curie temperature of the as-prepared films was almost 80 °C lower in comparison to the T_C_ of the rapidly quenched ribbon. There was also a significant decrease in the onset of the film crystallization temperature (T_x_). There were some assumptions that could be made for the understanding of observed behavior. Both as-quenched ribbon and as-prepared film have an amorphous structure with increased internal energy, i.e., they are in a metastable state. It is known that amorphous ribbon crystallization begins from the surface [33,34]. In the case of the film, the role of the surface increases substantially and surfaces may act in conjunction with the bulk crystallization provided by the influence of copper additions [35]. Furthermore, the film samples and samples in the form of the ribbons have a different initial structural state that follows from the XRD data analysis.

There are certain areas of X-rays coherent scattering (pre-segregation of the crystalline phase) in the as-prepared films, which could further influence the development of the crystallization process (see Figure 2 and Table 1). These reasons lead to a decrease in the temperature of the onset of crystallization. In turn, the decrease in the Curie temperature of the amorphous phase was a result of the size effect and, additionally, chemical and compositional disorder caused by the employed fabrication technique. The initial structural state of the films could also contribute to this effect.

The non-zero ferromagnetic signal at temperatures above the T_C_ of the amorphous phase and lower T_x_ values (inset Figure 1) indicates the presence in the as-prepared films a phase with a higher T_C_ than T_C_ of the amorphous phase. Perhaps it can be treated as a precursor of the crystalline phase or ultrafine “crystallites”: the higher signal intensity the greater volume of the phase. The XRD study shows the presence of coherent scattering areas of the slightly larger size of about 2 nm in the Mo-doped film. The pre-segregation of the crystalline phase, arising during the preparation process, causes a redistribution of elements inside the film that should lead to a change in the residual amorphous phase chemical composition and its magnetic characteristics such as the exchange interaction parameter, Curie temperature and saturation magnetization. The Curie temperature is mainly obeyed by the exchange interaction between atoms [36].

As it was suggested in [37,38] the Mo addition results in Fe–Fe interatomic distance decreased, which in turn decreases the exchange interaction between Fe atoms. The augment of the molybdenum content in the alloy composition simultaneously reduced the T_C_ of the amorphous matrix and increased the proportion of the crystalline phase. Comparison of the Fe_73.5_Nb_3_Cu_1_Si_13.5_B_9_ and Fe_72.5_Nb_1.5_Mo_2_Cu_1.1_Si_14.2_B_8.7_ shows that similar effect was also observed in the case of the films prepared by the method of ion-plasma sputtering.

Along with the decrease in the T_C_ a relative stabilization of the amorphous phase of the films should be expected. That is, the larger the resulting volume of ultrafine «crystallites» during film formation, the greater the observed decrease in the T_C_ of the amorphous phase. In addition, the larger the resulting volume of ultrafine «crystallites», the greater the expected increase in the temperature of the onset of crystallization, which was observed in a comparative analysis of the processes characteristic for the film shaped samples doped with Nb and NbMo.

Analysis of the data (Table 1) for Fe_73.5_Nb_3_Cu_1_Si_13.5_B_9_ films allowed us to conclude that the crystalline phase changed upon heating. It is known that silicon is highly soluble in iron. Si content increase leads to a decrease in the lattice parameter [39] and the formation of the bcc-Fe(Si) solid solution of substitution. After the heat treatments at 420 and 450 °C, the large grains lattice parameter was close to the value for bcc-Fe (a = 2.8664 Å). However, a somewhat smaller value indicates that the crystalline phase was predominantly a bcc-Fe(Si) solid solution with a silicon content in bcc-Fe of about 4 at. % (according to [40], which includes generalized data on the lattice parameter dependence on silicon content for single crystal, polycrystalline, and powdered FeSi samples). After the annealing at 500 °C, the lattice parameter seems to be slightly decreased, indicating the further Si content increasing in the bcc-Fe to the value of about 20 at. % [41]. The exact conclusion was difficult to make as for 500 °C annealed films the experimental error was higher due to the difficulty to take into account small contribution of the residual amorphous intergrain matrix. 

Thus, the presence of the ultrafine bcc-Fe grains with the size of about 1 nm in the initial state was shown in the Fe_73.5_Nb_3_Cu_1_Si_13.5_B_9_ films. It can be considered as a characteristic feature of the films obtained by ion-plasma sputtering. The film crystallization begins with the appearance of bcc-Fe solid solution nanocrystallites with the average size of about 20 nm. At the temperatures below 500 °C crystallization leads to a slight increase in the grain size but mainly by the increase of the volume fraction. The crystallization process rate significantly increased due to the activation of all existing crystallization centers as the heat treatment temperature rises. In such a way, heating to 500 °C leads to the structure consisting entirely of bcc-Fe(Si) grains with a size of about 20 nm and with the Si content of about 20 at. %.

The Fe_72.5_Nb_1.5_Mo_2_Cu_1.1_Si_14.2_B_8.7_ films XRD analysis during the heating (Figure 3 and Figure 4) showed a small decrease in the grain size and slower development of the crystallization process when the thickness of the sample changes from 200 to 100 nm. There are not a very high number of works devoted to the development of the crystallization process in FINEMET type thin film samples. However, it is possible to get some insight into the processes occurring in them based on the analysis of available data about the films synthesized by similar methods. Ref. [42] discusses the grain size decrease with the thickness decreasing in the films prepared by the magnetron sputtering. Besides with thickness decrease, an increase in internal stresses level was observed. Thus, based on the obtained results and the literature data, it can be assumed that in the 100 nm film a less ordered (in topological and chemical terms) structural state is realized, which is more resistant with respect to the development of the crystallization process.

**Figure 8:**
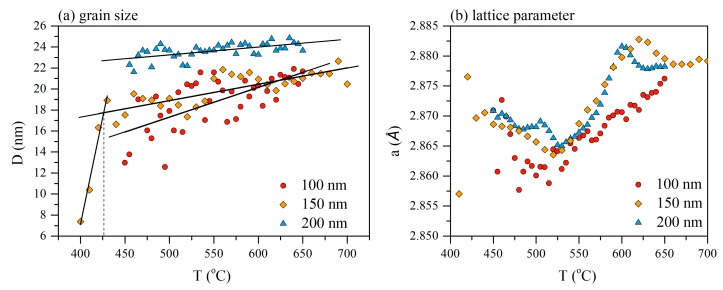
Grain size (**a**) and lattice parameter (**b**) dependence on temperature of the 100, 150 and 200 nm Fe_72.5_Nb_1.5_Mo_2_Cu_1.1_Si_14.2_B_8.7_ films.

Figure 8a shows that in the thicker films with the thicknesses of 150 and 200 nm a sharp grain growth was observed in the temperature range of 400–420 °C. With a subsequent increase in temperature, the grain size changed only very slightly as well as the lattice parameter does. From the results of the TDX analysis presented in Figure 3 and Figure 4, one could see the changes in the position of the main peak in the 2θ angle, reflecting the change in the interplanar spacing. The obtained dependences of the lattice parameter on the heating temperature of the films of different thickness are shown in Figure 8b. In the temperature range of 450–525 °C, a decrease in lattice parameters was observed. Since the main peak position on the XRD patterns corresponds to the bcc-Fe(Si) phase, the change in the lattice parameter could be interpreted due to the change in the chemical composition of the Fe(Si) solid solution.

Silicon is known as highly soluble in iron element forming a solid solution of substitution bcc-Fe(Si). At the temperature of above 525 °C the tendency changes and the lattice parameter increases. Without giving explicit reasons, the reliability of the change of the obtained dependence nature is confirmed by the results of [43,44] for the ribbons of a similar alloy with the difference that for ribbons changes occur at higher temperatures. However, the reason for the increase in the lattice parameter variation remains unclear. The origin of the temperature dependence of the lattice parameter change can be assumed as a consequence of the Si atoms ordering, which is well known for these alloys. Since silicon atoms diffuse into the grain through the grain boundary, the necessary concentration level first can be reached in the part of the grain adjacent to the boundary, where ordering probably begins, and then the process develops already due to the silicon contained in the grains. The Si concentration in the bulk of the grain decreases, and the lattice parameter of the solid solution may be increased.

Comparing the lattice parameters of the crystallites in the films with thicknesses of 100, 150 and 200 nm, it should be noted that 150 and 200 nm films demonstrate identical crystallization steps with the similar temperature dependence of the lattice parameter and grain size throughout the entire temperature range. Concerning these films, the crystallization process of a thinner film with a thickness of 100 nm is markedly different. Delayed crystallization and the formation of grains of smaller size and with a smaller lattice parameter are observed.

Figure 7 shows hysteresis loops for the studied films after the different heat treatments. The amorphous structure of the as-prepared films is characterized by large internal stresses. As can be seen from the hysteresis loops, after the heat treatment at 350 °C, a decrease in the coercivity of the 100 and 200 nm films was observed, indicating internal stress relaxation. The larger value of the thinner films coercivity is a consequence of the greater contribution of the surface roughness and the change in the domain walls energy density [45,46]. After annealing at 400 °C, the value of the coercivity is close to the coercivity value of the films annealed at 350 °C.

Based on the XRD study results, it can be assumed that at the temperature of about 400 °C the films are at the onset of crystallization stage, and their structure can be characterized by phase fluctuations. This structural instability can lead to variations in the coercivity value depending on the film sample. The noticeable differences in the hysteresis loops of the films are made oneself conspicuous after annealing at 450 °C. The coercivity increases for all films, but the shape of the loops of the films of different thickness undergoes changes. For the 100 nm films the hysteresis loop retains a rectangular shape, whereas, for the films with the thickness of 200 nm, there is a difficulty in the magnetization reversal process and a decrease in the residual magnetization, that is, the loop loses its squareness. In this case, the coercivity increase and the changes in the hysteresis loops shape are probably related to the crystallization of the film. Thus, a connection is found between the data of XRD analysis and hysteresis properties. In particular, a delayed crystallization with the film thickness decreasing is confirmed. After the heat treatment at the temperatures above 450 °C, the grain size of the Fe_72.5_Nb_1.5_Mo_2_Cu_1.1_Si_14.2_B_8.7_ is slightly above 20 nm, which apparently, as in the ribbons of similar compositions, exceeds the optimum crystallite size and causes deterioration of magnetic properties.

## 4. Conclusions

Comparison of the structural states of Fe_73.5_Nb_3_Cu_1_Si_13.5_B_9_ and Fe_72.5_Nb_1.5_Mo_2_Cu_1.1_Si_14.2_B_8.7_ thin films with the thickness of 200 nm shows that coarser grains were formed in the NbMo-doped case. In addition to the contribution of the preparation conditions and initially different structural states, partial replacement of niobium by molybdenum led to the grain size increase. It can be noted that thin films structural state of investigated alloys has several features. In the as-prepared films, the presence of the ultrafine crystallites with the size up to 2 nm was observed, and as the result of the heat treatment, the appearing crystalline grains contained both small and large size fractions. Crystallization began at the temperatures of 400–420 °C—substantially lower than that one observed for amorphous ribbons of similar composition alloys. In the temperature range under consideration, crystallization could be described as a sharp growth of bcc-FeSi grains up to about 20 nm. This behavior is explained by the presence of the crystalline phase precursors formed during the deposition of the films. As the temperature increased, the average grain size did not change, but the silicon content in the FeSi solid solution increased until the development of the ordering process. With the film’s thickness decrease, a tendency to a delay in crystallization was observed. The best magnetic properties of the Finemet type thin films were obtained after the stress relaxation. Crystallization led to a sharp increase in the coercivity and deterioration of the films soft magnetic properties.

## Figures and Tables

**Table 1:** Structural parameters of the 200 nm films of the Fe_73.5_Nb_3_Cu_1_Si_13.5_B_9_ alloy obtained after different heat treatment conditions.

State, Annealing Temperature (°C)	Annealing (min)	Grain Size (nm) for Fine Grains	The Fraction of Fine Grains (%)	Lattice Parameter of Fine Grains (Å)	Grain Size (nm) for Large Grains	The Fraction of Large Grains (%)	Lattice Parameter of Large Grains (Å)
As-prepared	0	<1	100	2.90(3)	-	-	-
420	30	<1	95	2.895(7)	16 ± 5	5	2.864(5)
450	10	<2	89	2.888(8)	16 ± 6	11	2.864(3)
	20	<2	78	2.880(8)	18 ± 6	22	2.865(3)
	30	<2	79	2.896(8)	20 ± 6	21	2.867(3)
500	30	<1	0	-	16 ± 5	100	2.85(2)

**Table 2:** Coercivity of the Fe_73.5_Nb_3_Cu_1_Si_13.5_B_9_ and Fe_72.5_Nb_1.5_Mo_2_Cu_1.1_Si_14.2_B_8.7_ thin films in the as-prepared state and after the heat treatment.

	Thickness (nm)			H_C_ (A/m)		
		AP	350 °C	400 °C	450 °C	500 °C
Fe_73.5_Nb_3_Cu_1_Si_13.5_B_9_	100	87.5	63.7	159	462	-
	200	23.9	47.8	15.9	318	660
Fe_72.5_Nb_1.5_Mo_2_Cu_1.1_Si_14.2_B_8.7_	100	294	175	175	1751	-
	200	87.5	47.8	350	732	875
